# Supplementary material for: Insect herbivores increase mortality and reduce tree seedling growth of some species in temperate forest canopy gaps
Source: PeerJ. 2017 Mar 21;5:e3102. doi: 10.7717/peerj.3102 (PMC5363256; doi:10.7717/peerj.3102)
Supplement: Supplemental Information 2 [file peerj-05-3102-s002.docx]

**Appendix 1 – Model Likelihoods and Descriptions**

We analyzed mortality, growth, and recruitment rates using Bayesian hierarchical models. Hierarchical models allow us to incorporate multiple levels of variation, enabling us to avoid pseudoreplication when necessary (described below, Gelman and Hill 2007, Zuur et al. 2009). Our statistical models are nearly identical in form to those of Beckage and Clark (2005), except that we treat gap age as a fixed effect rather than a random effect because we were specifically interested in the interactions between gap age and insecticide. By treating gap age as fixed and allowing the effects to vary randomly among plots, our model is identical to a repeated measures design in which individuals are sampled repeatedly throughout time.

Unfortunately, we could not use a paired design in our models because a paired design requires observations in both ‘Control’ and ‘Pesticide’ plots and this was not always possible. For example, in some gaps the ‘Control’ plot within a gap had measurements for *Acer rubrum* but the ‘Pesticide’ plot did not because *A. rubrum* was not present, rendering it impossible to calculate the difference between ‘Control’ and ‘Pesticide’ plots as required for a paired design. As a result, our design was unbalanced with differing numbers of observations for ‘Control’ and ‘Pesticide’ plots, but unbalanced data are not an issue for Bayesian models (Gelman and Hill 2007).

*1. Mortality Model*

To avoid pseudoreplication, we calculated the total number of dead individuals of each species in each plot. Plots in each year were therefore the unit of observation rather than individual germlings. The number of dead individuals of species *j* in plot *i* (*n_ij_*) was binomially distributed based on the total number of individuals *N_ij_* and a species-specific predicted mortality rate$\hat{p_{ij}}$:

$n_{ij}\sim\left( N_{ij},\hat{p_{ij}} \right)$

The predicted mortality was logit-transformed and a function of pesticide treatment and gap age:

$logit\left( \hat{p_{ij}} \right)=\beta_{0j}+\beta_{1j}{Pest}_{ij}+\beta_{2j}{Age2}_{ij}+\beta_{3j}{Age3}_{ij}+\beta_{4j}{{Pest}_{ij}\times Age2}_{ij}+\beta_{4j}{{Pest}_{ij}\times Age3}_{ij}$

where *Pest_ij_* is a dummy variable indicate whether the plot was a control (0) or sprayed with pesticide (1), *Age2_ij_* is a dummy variable indicating whether the gap was in year 2 (1) or not (0), and *Age3_ij_* is a dummy variable indicating whether the gap was in year 3 (1) or not (0). Coefficients were allowed to vary by species, and species-level parameters were drawn from a multivariate normal distribution:

$\left[ \beta_{0j} \right.,\beta_{1j},\cdots,\left. \beta_{4j} \right]\sim N\left( \Lambda,\Omega\right)$

where $\Lambda$ is a vector of the overall effects for each parameter and $\Omega$ is the covariance matrix of parameters.

*2. Growth Model*

We avoided pseudoreplication in analyses of growth rate by incorporating multiple levels of nesting (Zuur et al. 2009). First, our model predicted the average growth of a germlings within each species-plot-year combination:

$y_{ijk}\sim N\left( \hat{y_{ijk}},\sigma_{j}^{2} \right)$

where *y_ijk_* is the observed percent growth of species *j* in plot *i* in year *k* and $\sigma_{j}^{2}$ is a species-specific variance parameter*.* The fitted value was then a function of pesticide and gap age at the plot-level, as described above:

$\hat{y_{ijk}}=\beta_{0j}+\beta_{1j}{Pest}_{ij}+\beta_{2j}{Age2}_{ij}+\beta_{3j}{Age3}_{ij}+\beta_{4j}{{Pest}_{ij}\times Age2}_{ij}+\beta_{4j}{{Pest}_{ij}\times Age3}_{ij}$

By first modeling the mean growth of each species in each plot, and then treating pesticide and age effects as plot-level parameters, this model avoids treating individual seedlings as replicates (Zuur et al. 2009).

As described above, species-level parameters were drawn from a multivariate normal distribution:

$\left[ \beta_{0j} \right.,\beta_{1j},\cdots,\left. \beta_{4j} \right]\sim N\left( \Lambda,\Omega\right)$

*3. Recruitment Model*

Because yearly recruitment was highly variable and often absent for many species, we instead modeled the total number of recruits observed over the duration of the experiment for each species. This eliminated the effects of gap age and instead allowed us to model only the effect of pesticide. We treated number of recruits in each plot *i* for each species *j* as a Poisson distributed variable:

$r_{ij}\sim P\left( \hat{r_{ij}} \right)$

We used a log-link to model the predicted number of recruits in each plot as a function of pestidicde:

$log\left( \hat{r_{ij}} \right)=\beta_{0j}+\beta_{1j}{Pest}_{ij}$

and species-level parameters were drawn from a multivariate normal distribution:

$\left[ \beta_{0j} \right.,\beta_{1j}\left. \right]\sim N\left( \Lambda,\Omega\right)$

**References**

Beckage B, Clark JS (2005) Does predation contribute to tree diversity? Oecologia 143:458-469.

Gelman A, Hill J (2007) Data analysis using regression and multilevel/hierarchical models. Cambridge University Press.

Zuur AF, Ieno EN, Walker NJ, Saveliev AA, Smith GM (2009) Mixed effects models and extensions in ecology with R. Springer.
